# Modelling the impact of stunting on child survival in a rural Ugandan setting

**DOI:** 10.1186/s40795-018-0220-4

**Published:** 2018-03-27

**Authors:** John Bukusuba, Archileo N. Kaaya, Abel Atukwase

**Affiliations:** School of Food Technology, Nutrition and Bio-engineering, Makerere University, Kampala, Uganda

**Keywords:** Breastfeeding, Child mortality, IYCF, Stunting

## Abstract

**Background:**

Uganda ranks among the top 10 countries in the world for newborn and child mortality rates and among the top 34 for burden of stunting. This study was conducted to model the impact of stunting on child mortality in the southwest region of Uganda where the prevalence of stunting and child mortality are of great public health concern.

**Methods:**

The study was conducted in Buhweju district in the southwest region of Uganda. The study was cross-sectional involving use of a structured household questionnaire, focus group discussions and interviews with key informants in order to gather relevant information on infant and young child feeding (IYCF) and coverage of antenatal care (ANC) and vaccination programs. The survey of stunting, vaccination and ANC services covered 221 children aged 6–59 months while the assessment of IYCF practices covered 83 children aged 0–23. The Lives Saved Tool (LiST) was used to estimate the impact of stunting on child mortality and cases of stunting averted.

**Results:**

The study findings indicated that only 33% of the mothers had knowledge of optimal IYCF and 39% of the sampled children were exclusively breastfed. The majority of the mothers (57%) breastfed their children for less than 2 years and only 31% of the mothers practiced appropriate introduction of complementary foods at 6 months. Only 17% of the 0–23-month-olds received a good standard of IYCF. Only 37% of the mothers reportedly attended ANC 4 times or more during pregnancy and among children aged 6–59 months, only 28% were fully vaccinated. The high impact LiST model estimated that 1297 children under 5 years would be saved and 24,850 cases of stunting averted in the study district.

**Conclusions:**

The study concludes that IYCF practices and coverage of expanded programme on immunization (EPI) and ANC in the study population are sub-optimal thus the high prevalence of stunting and child mortality in the region. LiST demonstrated that prevention of stunting would reduce child mortality in rural Uganda. Therefore, increased investment in cost-effective child survival interventions targeting rural areas of Uganda would have a significant impact on stunting and child mortality.

## Background

Uganda ranks among the top 10 countries in the world for newborn and child mortality rates [1] and among the top 34 for burden of stunting [2–6]. In Uganda, the neonatal mortality rate (NMR) is at 27 per 1000 live births, the infant mortality rate (IMR) is at 43 per 1000 live births and the under-five mortality rate (U5MR) is at 64 per 1000 live births [3, 4]. One in every 25 Ugandan children dies before their first birthday, and one in every 15 children dies before their fifth birthday. The southwest region has the second highest IMR in Uganda, at 76 per 1000 live births, and a U5MR of 128 per 1000 live births [4]. Similarly, the prevalence of stunting in southwest region is the third highest in Uganda, at 42% [4].

Uganda did not meet its Millennium Development Goal (MDG) targets to reduce the U5MR to 56 deaths per 1000 live births and the IMR to 31 per 1000 live births in 2015, and is likely to fail to meet the national targets to reduce the U5MR from 90 to 51 per live births by 2020 [7]. This could be attributed to uneven sub-national progress on child mortality and stunting. The persistent high U5MR may also partly be attributed to the low coverage of preventive evidence-based interventions known to be cost-effective in reducing child mortality, for example the expanded programme on immunization (EPI), optimal infant and young child feeding (IYCF) practices and antenatal care [1, 3, 4, 8, 9].

There is evidence that providing vaccines to infants and children against tuberculosis, polio, whooping cough, diphtheria, tetanus, measles, hepatitis B and haemophilus influenzae reduces infant and under-five mortality rates [3, 4, 9]. Similarly, sub-optimal IYCF practices are a major risk factor for child mortality and stunting. Studies have shown that up to 19% of child deaths can be prevented through optimal IYCF practices [10, 11]. Therefore, optimal IYCF practices can have the single largest impact on child mortality of all preventive interventions [12–14].

Several studies have used the Lives Saved Tool (LiST) in developing countries to model the impact of evidence-based interventions and stunting on child survival at national level [15–20]. However, the impact of stunting on child survival has not been formally modelled at sub-national level particularly in resource-limited settings where the burden of child mortality is highest. Our study therefore used LiST to model the impact of stunting reduction on child mortality in the southwest region of Uganda where child mortality and stunted growth are of great public health concern [1, 3, 12]. We also assessed the coverage of programs (antenatal care, EPI and IYCF practices) that have been shown to have high impact on prevention of stunting and child survival [12, 15, 16, 20, 21].

## Methods

This study was undertaken as part of a larger project evaluating the risk factors and associated cost of preventing childhood stunting in southwest Uganda. The results on prevalence and risk factors of stunting have been published elsewhere [22].

### Study site

The study was conducted in Buhweju district, which is located in southwest Uganda. The district was chosen because it is one of the districts in south western region where previous localized nutrition surveys have reported a high prevalence of stunting in a region deemed to be food secure [22, 23]. The district consists of one county (Buhweju) and eight sub-counties (Bihanga, Burere, Karungu, Rwengwe, Bitsya, Nsiika Town Council, Engaju and Nyakishana). The villages where data was collected were randomly selected from a list of all villages in the eight sub-counties.

### Study design and data collection methods

The current study was cross-sectional and was conducted between September and October 2016. We developed and used a structured household questionnaire and we ran focus group discussions and interviews with key informants. The study was conducted alongside a survey of nutrition and stunting [22]. Both studies were carried out in 32 randomly selected villages in Buhweju district. The probability proportional to size (PPS) method was used to randomly select the study villages from the 216 villages in Buhweju district. Systematic random sampling was then used to select eight households for the nutrition and stunting survey (which included assessment of ANC and EPI coverage) from each of the villages [22]. All selected households with children aged 0–23 months were included in the study of IYCF practices.

The interviewers underwent a 2-day training course to standardize the assessment procedure and ensure that high-quality data were collected. The training included a 1-day class and a field pre-test on the second day. The survey questionnaires were pre-tested to ensure that: 1) the questions were well formulated, easily understood and ethically acceptable, 2) the order of the questions was logical and 3) the information yielded addressed the study objectives [24].

### Sample size calculation

The sample size calculation for the larger study that assessed the prevalence of stunting and its risk factors has been published elsewhere [22]. The sample size for the study of IYCF practices among children under 2 years was determined using the Infant and Child Feeding Index (ICFI) approach. This approach was used because the World Health Organization (WHO) recommends that, in general, IYCF indicators should only be used in large-sample surveys (e.g., Multiple Indicator Cluster Surveys or Demographic and Health Surveys) as they are not suitable for surveys at sub-national levels, where small-sample surveys are often used [25–28]. The ICFI approach was developed as an alternative approach to assess IYCF practices using small-sample surveys. The ICFI approach is, in large part, a development of earlier work undertaken by the International Food Policy Research Institute and the Food and Nutrition Technical Assistance Project [28, 29].

The sample size for the ICFI study was calculated based on the formula below:

$$ {n}_{ICFI}=\frac{23-6+1}{59-6+1}\times {n}_{Nutrition\ survey} $$

*n*_*ICFI*_ = Sample size for the ICFI survey.

*n*_*Nutrition survey*_= Sample size for the survey on nutrition and stunting.

Three indicators were used for scoring IYCF practices: breastfeeding, dietary diversity and meal frequency (Table 1). Seven food groups were used to measure dietary diversity (Cereals, roots and tubers; legumes and nuts; dairy products; meat and fish; eggs; vitamin-A rich fruits and vegetables; and other fruits and vegetables) [26, 27].

$$ ICFI= Breastfeeding\ indicator+ Dietary\ Diversity\ indicator+ Meal\ Frequency\ indicator $$

**Table 1:** ICFI scoring scheme for age-appropriate feeding practices

Indicator (based on 24-h recall periods)	Age group (months)					
	6–8		9–11		12–23	
	Response	Score	Response	Score	Response	Score
Breastfed	Yes	+ 2	Yes	+ 2	Yes	+1
Number of food groups	1	+1	1 or 2	+1	2 or 3	+1
	≥2	+ 2	≥3	+ 2	≥4	+ 2
Meal frequency per day	1	+1	1 or 2	+ 1	2	+1
	≥2	+ 2	≥3	+ 2	3	+ 2
					≥4	+ 3

Children aged 0–5 months who were exclusively breastfed and those aged 6–23 months with ICFI scores of ≥6 were classified as receiving a good standard of IYCF [28, 29].

### Focus group discussions (FGDs) and interviews with key informants

**Fig. 1:**
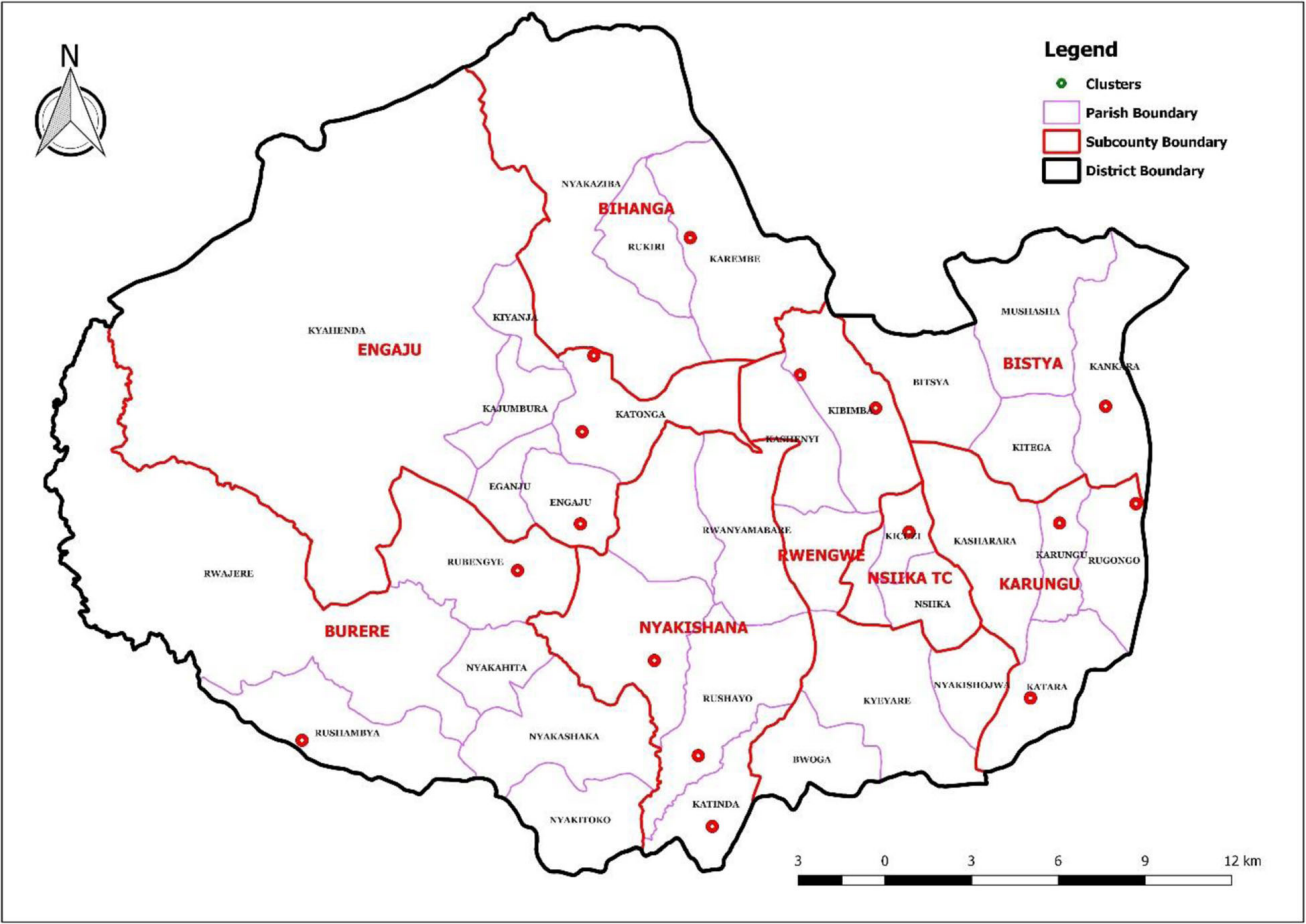
Villages where FGDs and interviews with key informants were conducted

A total of 16 focus group discussions and 16 interviews with key informants on IYCF knowledge and practices, EPI and ANC services were conducted in 16 of the 32 villages that were selected for household interviews (Fig. 1). For each focus group discussion, 10–15 participants were purposively selected. These mainly included village leaders, health workers and mothers of children under 5 years of age, and each focus group discussion lasted about 1–2 h. Interviews with individual key informants were carried out to obtain specific information from individuals that were selected due to their knowledge of ANC services, EPI and IYCF practices.

### Expanded programme on immunization

Information on vaccination coverage was obtained in two ways – from vaccination (child health) cards and from mothers’ recall [3, 4, 9]. If a vaccination card was available, the interviewer recorded onto the questionnaire the dates of each vaccination received by the child. However, where the child never received a vaccination card or if the vaccination was not recorded, the vaccination information for the child was based on the mother’s recall [3, 4, 9].

### Lives saved and number of cases of stunting averted

The Lives Saved Tool (LiST) [16–20] which is incorporated into the OneHealth software was used to estimate the impact of stunting reduction on child mortality and cases of stunting averted in two scenarios – a) halving the prevalence of stunting by 2040, b) zero stunted children in Buhweju district by 2040. The modelling period was set at 2017–2040 to align with the Uganda vision target of zero stunted growth by 2040 [7]. The number of cases of stunting averted was calculated using the difference between the estimated stunting prevalence in the target year with the prevalence in the base year. This percentage change was then multiplied by the population projection of children under 5 years of age in Buhweju district.

### Data management and analysis

In each village, the household questionnaires were checked for completion at the end of each day and every evening. Data clerks entered the responses to the questionnaires received from the various villages into a database. The recordings of focus group discussions and interviews with key informants were transcribed, entered into a Microsoft Excel database and systematically coded. Data entry, cleaning and analysis were carried out using Statistical Package for Social Sciences (SPSS) version 21 and for all tests of associations, *p*-value ≤0.05 was considered statistically significant.

Bivariate analyses were conducted using chi-square statistics to examine factors associated with optimal IYCF practices and access to child survival interventions. The variables included in these analyses were: early initiation of breastfeeding within 1 h, use of pre-lacteals, exclusive breastfeeding, appropriate introduction of solid, semi-solid or soft foods, continued breastfeeding to 2 years of age, minimum dietary diversity (MDD), minimum meal frequency, minimum acceptable diet (MAD), EPI, vitamin A supplementation, deworming, antenatal care and skilled attendant during childbirth. We used the results of stunting prevalence obtained from the survey of nutrition and stunting [22] to model in LiST the impact of stunting reduction between 2017 and 2040 on child mortality.

## Results

### Socio-demographic characteristics of the study population

The socio-demographic characteristics of the study population has been published elsewhere [22]. Briefly, the survey of nutrition and stunting covered 256 households and 221 children aged 6–59 months while the study of IYCF practices covered 82 households and 83 children aged 0–23 months, of which 53% were male and 47% female. The average household size in both surveys was five people. The majority of the mothers (70%) had education levels of up to primary school and the average age was 29 years.

### Breastfeeding knowledge and practices

Mothers’ knowledge about early initiation of breastfeeding (within the first hour) and exclusive breastfeeding among children less than 6 months were found to be 84% and 57% respectively. Accordingly, the practice of early initiation of breastfeeding within an hour after birth was reportedly 73% while only 39% of the children less than 6 months were exclusively breastfed. Good knowledge about the benefits and timing of initiation of breastfeeding was significantly correlated with the actual practice and less use of pre-lacteal foods and fluids.

Continued breastfeeding to 2 years or beyond was reported among only 43% of the mothers despite 71% of them having good knowledge of the recommended breastfeeding duration. Knowledge of the recommended breastfeeding duration was significantly (*p < 0.05*) associated with the actual practice. About 27% of the mothers reported that their children were fed from a bottle with a nipple at the time of the survey. Sub-optimal breastfeeding was mainly attributed to use of pre-lacteal foods and fluids (51%), discouragement (23%), early pregnancy (37%) or insufficient breastmilk (26%).

Most participants in the focus group discussions and key informant interviews agreed with the recommendation that initiation of breastfeeding should be immediate because of the health and nutrition benefits of colostrum. However, some also said that giving pre-lacteals (mainly water and herbs) is a common practice. The main reason given for giving pre-lacteals was to clean the throats of the newborns. The participants also agreed that infants should be exclusively breastfed for 6 months. However, the participants indicated that exclusive breast feeding was not a common practice in the study population because of insufficient breastmilk, preoccupation with farming activities or the human immunodeficiency virus (HIV)-positive status of the mothers. Therefore, some participants thought that exclusive breastfeeding should only be practiced for 3 months.

Although most mothers who participated in focus group discussions agreed with the recommendation that breastfeeding should be continued to 2 years and beyond, many said that this practice was rare in the study population because of frequent pregnancies and the belief that children tend to refuse breastmilk even before 1 year of age.

### Complementary feeding knowledge and practices

The proportion of mothers who appropriately introduced complementary foods to their infants at the age of 6 months was only 31%, despite most of the mothers (56%) understanding the appropriate age for introduction of complementary foods. Bivariate analysis showed that mothers who had knowledge regarding the appropriate age for introduction of complementary foods were significantly (*p* < 0.05) more likely to start complementary feeding at 6 months.

When mothers were asked which foods constitute good complementary foods, majority reported plantain (79%), cereals and all cereal products (73%), legumes and nuts (41%), dark green leafy vegetables (26%), roots and tubers (21%), dairy products (17%), eggs (14%), vitamin A-rich fruits and vegetables (7%) and meat and fish (6%). Only 10% of the mothers could correctly identify at least four food groups. Mothers reported that the main sources of information on IYCF were interpersonal communication (49%), health centers (26%) and mass media (23%).

In the focus group discussions and key informant interviews, when asked about the types of complementary foods that should be given to children from 6 months of age, most participants mentioned only two food groups (cereals, roots and tubers and dark green leafy vegetables). Participants also reported that there were no IYCF programs in their villages and no structured dissemination of the IYCF information. The main sources of information were interpersonal communication, health workers at health facilities and village health teams.

**Fig. 2:**
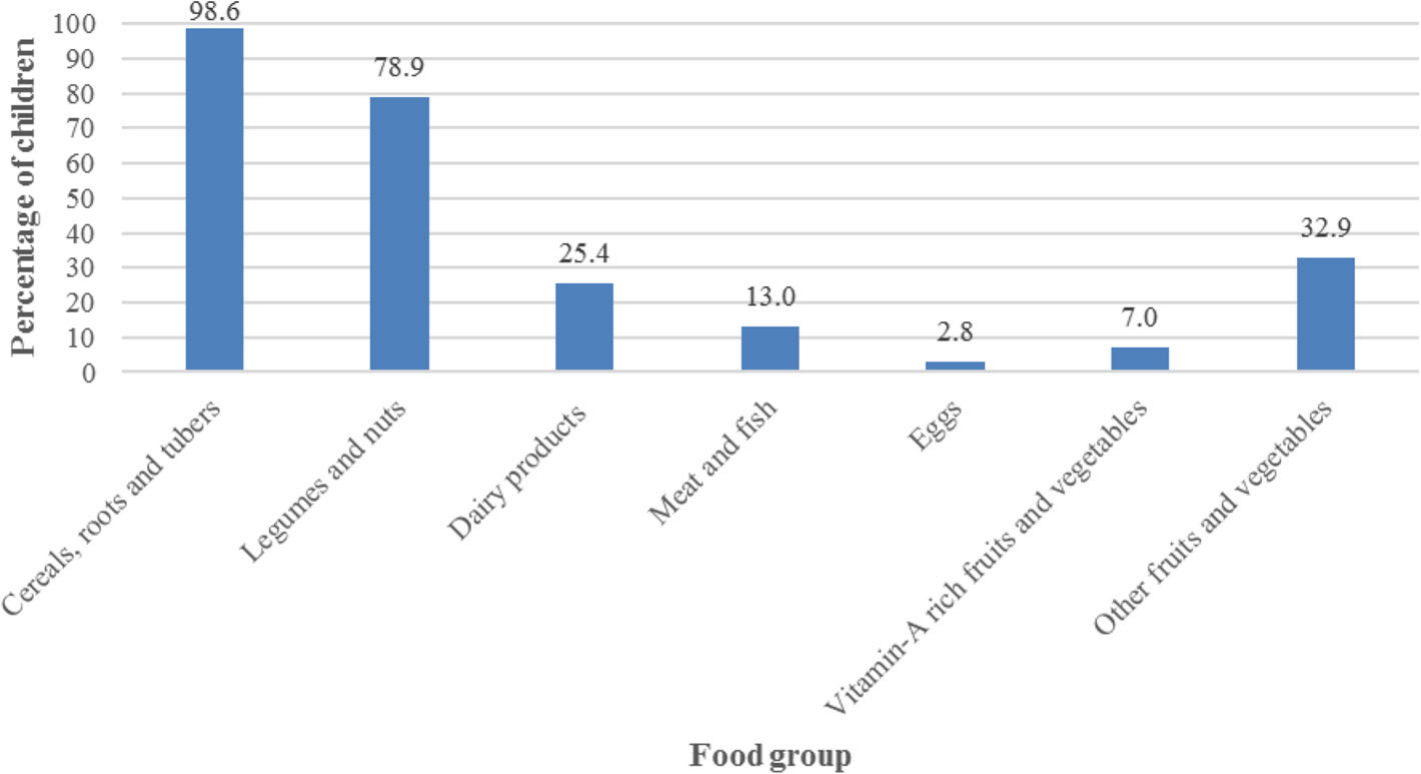
Percentage of children aged 6–23 months who ate specific food groups

Results presented in Fig. 2 show that complementary foods given to children aged 6–23 months were mainly from two food groups (cereals, roots and tubers; legumes and nuts). Most children hardly consumed animal protein (13%), eggs (3%), dairy products (25%) and vitamin-A rich fruits and vegetables (7%). Therefore, only 16% of the children received the recommended minimum dietary diversity (proportion of children aged 6–23 months who receive foods from four or more food groups according to WHO [26, 27]) and only 12% of them received both a diverse diet and adequate number of meals.

### Infant and Child Feeding Index (ICFI)

The study findings indicated that only 33% of the mothers correctly understood the meaning of optimal IYCF. The mean ICFI score was 3.5, and only 17% of the children aged 0–23 months were classified as receiving a good standard of IYCF. Children born to mothers who understood the meaning of optimal IYCF were significantly more likely to receive a good standard of IYCF (*p* < 0.05). The correlation between ICFI and age of the child showed that the ICFI score was significantly higher (*r* = − 0.43, *p* < 0.05) among young children. This implies that young children were more likely to be classified as receiving a good standard of IYCF compared to older children (i.e., the older the child gets, the poorer their ICFI score).

### Antenatal care (ANC) coverage

The results indicated that majority of the mothers (89%) reportedly attended ANC at least once during pregnancy but only 37% of them reportedly attended ANC 4 times or more. Most of those who attended ANC (79%) reportedly received iron-folate supplements and a Tetanus Toxoid (TT) vaccine (82%). However, only 46% of the expectant mothers received Tetanus Toxoid (TT) vaccine at least 2 times or more. As expected, mothers who attended ANC 4 times or more were significantly (*p < 0.05*) more likely to also receive Tetanus Toxoid (TT) vaccine at least 2 times or more. Deworming tablets were taken by 63% of the expectant mothers and 76% of them also received intermittent preventive treatment for malaria.

### Coverage of EPI, vitamin a supplementation and deworming

Among children aged 6–59 months, only 28% were fully vaccinated (eligible child received BCG, 3 doses of Polio, 3 doses of DPT and measles), 45% received vitamin A supplementation and 43% were dewormed within 6 months preceding the survey (Table 2). Children who were born in a health facility were 3 times more likely to be fully vaccinated than those whose birth place was home or by traditional birth attendant (*χ*^*2*^ = 10.07; OR = 2.6, 1.4–4.9, *p < 0.01*).

**Table 2:** Coverage of EPI, vitamin A supplementation and deworming

Intervention	Card			Recall			Card or recall		
	No.	%	95% CI	No.	%	95% CI	No.	%	95% CI
BCG	116	52.5	45.3–59.3	84	38.0	31.7–44.8	200	90.5	86.4–94.1
Polio	72	32.6	26.7–38.5	21	9.5	5.9–13.6	93	42.1	35.3–48.4
DPT-HepB-Hib	110	49.8	43.0–56.1	44	19.9	14.9–25.8	154	69.7	63.3–75.6
Measles	112	52.1	45.1–58.6	75	34.9	28.8–41.9	215	87.0	82.8–91.2
Full Vaccination	54	25.1	19.5–31.2	6	2.8	0.9–5.1	60	27.9	21.9–34.4
Vitamin A supplementation	27	12.2	7.7–16.7	73	33	26.7–39.8	100	45.2	38.5–51.6
Deworming				94	42.5	36.2–49.3			

### Impact of stunting reduction on child mortality

**Fig. 3:**
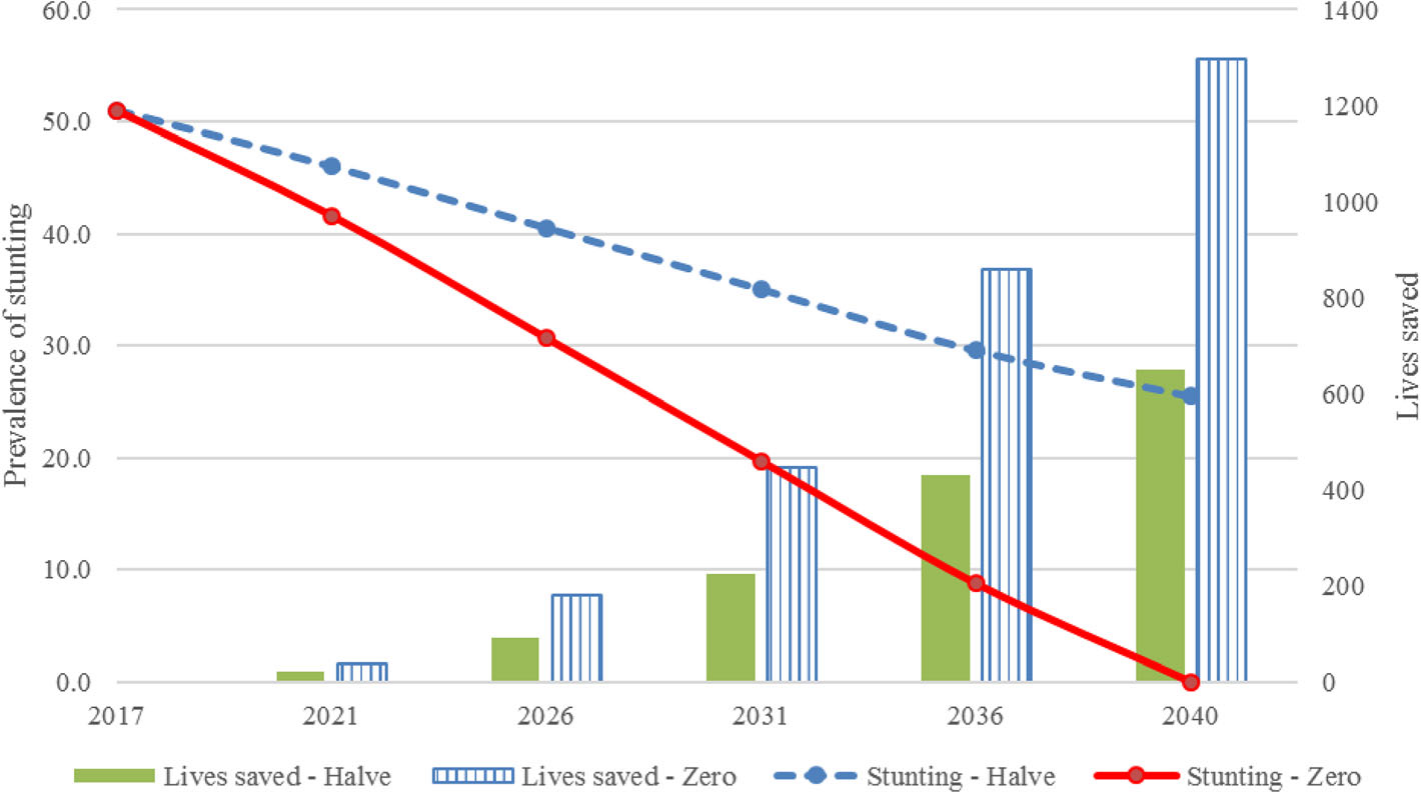
Children saved in the two scenarios

**Fig. 4:**
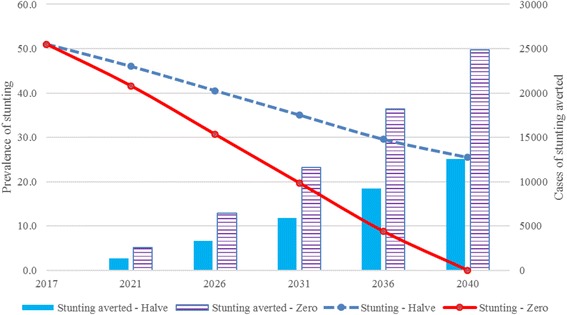
Number of cases of stunting averted in the two scenarios

The estimates of lives saved and cases of stunting averted based on the two scenarios are presented in Figs. 3 and 4. In the first scenario (halve the prevalence of stunting by 2040), LiST estimated that 650 children under 5 years would be saved and 12,571 cases of stunting averted (Figs. 3 and 4). In the second scenario (reduction of stunting to zero by 2040), it was estimated that 1297 children under 5 years would be saved and 24,850 cases of stunting averted (Figs. 3 and 4).

## Discussion

The inclusion of stunting and child mortality targets in the Uganda vision 2040 aimed at reducing stunted growth from 31% to 0% and U5MR from 90 to 8 per 1000 live births by 2040 [7] creates an enabling environment and opportunity to sustain the momentum on improving child survival in Uganda. This creation of an enabling environment has progressively been translated into policy and financial support by the government to increase the coverage of cost-effective child survival interventions [7, 9, 11]. However, there is still inequitable coverage of these interventions particularly in southwest districts like Buhweju where stunting and child mortality are of great public health concern [4, 22]. Our study conducted in southwest region shows that IYCF knowledge and practices in rural Uganda remain very poor. Similarly, although EPI and ANC are known to be among the most cost-effective interventions [15, 16, 18], the coverage of these programs in the study population was very low.

The World Health Organization recommends initiation of breastfeeding within an hour of birth, exclusive breastfeeding of infants up to 6 months of age and continued breastfeeding until the children are 2 years or older [13, 30]. However, in the current study a large proportion of mothers neither understood the meaning of optimal breastfeeding nor practiced it. Majority of the mothers also reported use of pre-lacteals (mainly water) before the commencement of breastfeeding. The use of pre-lacteals denies these infants the protective effect of colostrum associated with early initiation of breastfeeding. This practice therefore exposes the infants to increased risk of infections, stunting and mortality as other studies have also reported [31, 32]. There is global evidence showing that initiation of breastfeeding within 24 h of birth is associated with 44–45% reductions in all-cause and infection-related neonatal mortality [11]. Pre-lacteal feeding also has many implications for exclusive breastfeeding and resulting consequences since by definition, these children are not exclusively breastfed. Similar to our study findings in which mothers reportedly used pre-lacteals to clean the throats of the newborns, a study that assessed the factors associated with pre-lacteal feeding in the rural population of northwest Ethiopia reported that the practice was largely associated with poor knowledge of IYCF [33].

When children are given complementary foods from the age of 6 months, the foods should be adequate to support growth (i.e., an adequate quantity of food for each meal and number of meals per day) and have the right consistency, nutrients and energy density. However, most mothers in the study area did not practice timely introduction of complementary foods and majority of the children did not receive a minimum acceptable diet. Sub-optimal IYCF practices are common in Uganda [3, 4, 34], which is consistent with the findings of our study in which majority of the children aged 0–23 months were classified as not receiving a good standard of IYCF. According to the UBOS [3, 4], although almost all children (98%) in the Uganda Demographic and Health Survey (UDHS) were breastfed at some point, only half (53%) were breastfed within an hour of birth and only 66% of children who were aged 0–5 months at the time of the survey were exclusively breastfed. By 4–5 months of age, the proportion of exclusively breastfed children dropped to only 43%. The proportion of 2-year-olds who were breastfed was only 50% [3, 4]. In addition, among children aged 6–23 months, 13% were fed the recommended minimum dietary diversity, 45% received the recommended minimum number of meals per day and only 14% received the minimum acceptable diet [3, 4].

The lower ICFI scores particularly among older children found in this study could be attributed to the lack of knowledge on optimal IYCF and adequate skilled support at health facility and community levels, poor complementary feeding practices including low meal frequencies (since most mothers were reportedly preoccupied with farming activities) and non-responsive feeding as reported during FGDs. Therefore, it is not surprising that only 12% of the children received the recommended minimum acceptable diet. A study in Cambodia also reported higher child feeding index (CFI) scores among young children compared with old ones [21].

Sub-optimal IYCF practices and low coverages of EPI and ANC are associated with increased levels of stunted growth and child mortality [2, 11, 35–37]. The consequences of sub-optimal IYCF practices and low coverage of EPI and ANC services are evident in the persistently high prevalence of stunting and child mortality rates in Uganda, as these practices compromise child growth and development. Therefore, it is not surprising that Uganda is among the developing countries with the largest proportions of stunted children and high burden of child mortality [1, 3, 4]. In Uganda, one out of every three (29%) children under 5 years are stunted and 167,000 children under 5 years die every year [1, 3, 4]. Children born in rural areas, with uneducated mothers or among households in the lowest wealth quintile are more likely to be stunted or die before their fifth birthday [1, 4]. The southwest region recorded the third highest proportion (42%) of children who were stunted and second highest U5MR of 128 per 1000 live births out of all the regions in Uganda [4]. The recent nutrition survey conducted in Buhweju district showed that very little progress has been made in reducing the prevalence of stunting which is currently at 51% [22]. Therefore, the findings of this study suggest that the high prevalence of stunting and child mortality in Buhweju district could be largely attributed to low coverage of child survival interventions (including sub-optimal IYCF practices and low coverage of EPI and ANC services).

In Uganda, although 97% of pregnant women attend their first antenatal visit, only 60% complete the World Health Organization (WHO) recommended minimum of four antenatal visits [1, 3, 4]. Much lower ANC attendance compared to the national and southwest region averages were registered in Buhweju district. This could be attributed to the low availability and access to antenatal care in the district. Most participants in FGDs and interviews with key informants also reported that the low quality of ANC services (understaffing, poor counseling services and poor client-provider relations) in facilities where it is provided has partly contributed to the poor attendances. It is well known that on average, less than one-quarter of facilities in Uganda have all essential equipment and supplies for basic ANC [1, 37]. In addition, although vaccination coverage in Uganda has improved over the last 10 years [1, 3, 4], majority of the children under 5 years in Buhweju district were not fully vaccinated. Low vaccination coverage exposes the children to an increased risk of dying from preventable diseases. Previous reviews of the main factors associated with low vaccination coverage in Uganda and other developing countries found these to be: maternal education, maternal age, exposure to media, maternal healthcare utilization, health staff attitudes and practices, reliability of health services, parents’ practical knowledge of vaccination, fear of side effects, conflicting priorities, parental beliefs and immunization plan [9, 38]. These risk factors for low vaccination coverage are similar to the findings of our study in which access to health services and ANC contributed to higher vaccination coverage.

In the study population, there were also many missed opportunities along the continuum of care (from gestation to 5 months of age) for the delivery of nutrition education regarding optimal IYCF and communication of information on EPI. For example, in our study a small proportion of the mothers reported receiving information on IYCF from health care providers. The low consumption of animal sources of protein (eggs, dairy products, meat and fish), as evidenced in the current study, is likely to be another major contributor to the high levels of stunted growth and mortality among children in Buhweju district. A study in Malawi that analyzed blood samples from 313 children aged 12–59 months (with and without stunting) found lower serum concentrations of all nine essential amino acids in the stunted children compared with non-stunted children [39].

In modelling the impact of stunting on child survival in a rural setting, our study demonstrates that reducing the prevalence of stunting to zero would result in the highest impact on child mortality – saving more lives and averting more cases of stunted children. It is therefore crucial to inform decision-makers on the need to support the prioritization of interventions aimed at reduction of stunting and advocacy for increased funding of maternal and child survival interventions. There is already evidence that LiST identifies priority areas for child health investment based on modelling the impact of evidence-based interventions at varying levels of coverage [15–18, 20]. Although LiST has been mainly used to model the impact of scaling up maternal, newborn and child health intervention on child mortality [15–20], in our study, we presented the impact of reducing the prevalence of stunting on child survival in a rural setting. It is therefore important to note that the approach used in our study, models the impact of reducing stunted growth on child mortality but does not provide details on how stunting reduction can be achieved.

## Conclusions

The study concludes that IYCF practices and coverage of EPI and ANC in the study population are sub-optimal thus the high prevalence of stunting and child mortality in the region. LiST demonstrated that prevention of stunting would reduce child mortality in rural Uganda. Therefore, increased investment in cost-effective child survival interventions targeting rural areas of Uganda would have a significant impact on stunting and child mortality. The translation of messages regarding nutrition education into actual practice focusing on increasing exclusive breastfeeding and consumption of animal sources of protein would boost linear growth in children under 2 years of age and reduce mortality. Social and behavior change communication (SBCC) designed to promote optimal IYCF, EPI and ANC should target multiple delivery channels and also address reported barriers.

## Abbreviations

ANC: Antenatal care
EPI: Expanded programme on immunization
FGDs: Focus group discussions
HIV: Human immunodeficiency virus
ICFI: Infant and child feeding index
IMR: Infant mortality rate
IYCF: Infant and young child feeding
LiST: Lives saved tool
MDG: Millennium development goal
NMR: Neonatal mortality rate
PPS: Probability proportional to size
SBCC: Social and behavior change communication
SPSS: Statistical package for social sciences
TT: Tetanus toxoid
U5MR: Under-five mortality rate
UDHS: Uganda demographic and health survey
WHO: World Health Organization
